# Forensic age assessment of late-term bovine fetuses

**DOI:** 10.1186/s13028-023-00691-0

**Published:** 2023-06-24

**Authors:** Jørgen Steen Agerholm, Maria Dahl, Mette Herskin, Søren Saxmose Nielsen

**Affiliations:** 1 Department of Veterinary Clinical Sciences, University of Copenhagen, Højbakkegård Allé 5A, 2630 Taastrup, Denmark; 2 Department of Animal and Veterinary Sciences, Aarhus University, 8830 Tjele, Denmark; 3 Department of Veterinary and Animal Sciences, University of Copenhagen, Grønnegårdsvej 8, 1870 Frederiksberg C, Denmark; 4 Present Address: Skovbjerg DyrlægeTeam, Grenevej 21, 6752 Glejbjerg, Denmark

## Abstract

**Background:**

Transporting pregnant cattle that have passed 90% or more of the expected gestation period (G90 threshold) is prohibited within the European Union. Therefore, there is a need to determine whether this threshold has been exceeded in late-gestation cows sent to slaughter. The aim of this study was to evaluate fetal parameters’ reliability for use in forensic age assessment of late-term Holstein fetuses.

**Results:**

Analysis of the gestation length of 2734 Holsteins that calved with a single liveborn fetus revealed a median gestation length of 278 days with 99% of parturitions occurring between day 261 and 290, corresponding to G90 thresholds of 235 and 261 days, respectively. The association between gestation length and neonatal body weight had an R^2^ of 0.27. The influence of fetal sex and cow parity on gestation length was ± 2 days. The eruption of incisor and canine teeth was assessed in preterm calves delivered by caesarean section (n = 52) and full-term neonatal calves (n = 54). Statistical analysis of tooth eruption data showed a statistically significant variation in fetal age at tooth eruption.

**Conclusions:**

Defining the G90 threshold for a cow not having reached parturition is challenging. Body weight was not found to be a reliable parameter for identifying fetuses beyond the G90 threshold. Statistical analysis of the association between fetal age and eruption through the gingival mucosa of incisor and canine teeth revealed significant variation, making tooth eruption a challenging parameter to use in forensic cases. Assessment of the evaluated parameters, therefore, cannot be considered a scientifically validated method to conclude definitively and beyond reasonable doubt whether or not a given fetus has passed the G90 threshold.

**Supplementary Information:**

The online version contains supplementary material available at 10.1186/s13028-023-00691-0.

## Background

A European Union council regulation prohibits (although with certain exceptions; [1]) the transport of pregnant cattle that have passed 90% or more of the expected gestation period (G90 threshold). According to the legislation, late-term pregnant cattle are considered too vulnerable to be transported, and if they start to calve during transport, both the dam and its offspring are at risk of severely compromised welfare. Despite this prohibition, late-term pregnant cattle may be transported—intentionally or unintentionally, thus resulting in a need for veterinary authorities to clarify whether or not the animal has passed the G90 threshold as part of the forensic enforcement of legislation.

The length of the gestation period for cattle is not a well-defined number of days, so an accurate G90 threshold for a specific individual can only be calculated with absolute certainty when both the breeding date and the date of parturition are known, i.e., it can only be determined retrospectively once parturition has occurred and with a valid breeding date. If the date of breeding is not available, an accurate calculation of the gestation length (GL) at slaughter will not be possible.

It is therefore not possible to establish an accurate gestation length for pregnant cattle transported to slaughter and arriving before the initiation of parturition. To overcome the challenge of calculating the G90 threshold for prepartal cattle, the EU regulation refers to an “expected gestation period” as a basis for the calculation. However, expected GL is not a scientifically well-defined period as it depends on many variables such as breed, number of fetuses, parity and season [2–5], and it varies among individuals even within a group of otherwise comparable animals. This is of particular significance when the GL is close to the G90 threshold, as even a small number of days of uncertainty in the calculation can, in principle, differentiate legal from illegal transport.

Forensic handling of cases of late-term pregnant cattle sent for slaughter is challenging for the above-mentioned reasons, unless the animal is already showing signs of imminent calving when inspected by the authorities, e.g., in connection with *ante mortem* inspection. Suspected violation of the transport regulation is often not raised until the abdominal organs have been removed from the carcass and a fully developed fetus is observed upon inspection of the uterus (JSA, personal observation). In cases where a specific breeding date is not available, the GL can only be determined by fetal characteristics. However, validated data on fetal development characteristics of late-term bovine fetuses are sparse [6] and establishing forensic evidence for fetal age is therefore challenging in such cases.

The overall aims of the present study were to provide scientifically validated data for potential use in forensic age assessment of late-term Holstein fetuses and to determine their usefulness in identifying fetuses that have passed the G90 threshold. These were achieved by: (1) providing detailed data on variation in the GL and body weight (BW) of late-term fetuses and neonatal calves; (2) estimating the correlation between GL and neonatal calf BW; (3) determining the order and progression of the incisor teeth and canine teeth erupting through the gingival mucosa, and (4) evaluating the use of BW and tooth eruption to identify fetuses that have passed the G90 threshold.

## Methods

The objectives were addressed using data from three different sources. Data from a research herd were used to analyse variation in GL and to obtain BW data on neonatal calves. Data from elective caesarean section training sessions were used to obtain data on the BW of late-term fetuses and tooth eruption, while data from a study of neonatal calves in a dairy herd were used to obtain data on tooth eruption in full-term calves.

### Gestation length and body weight of neonatal calves

Data on GL, parity, sex and BW at delivery from Holstein cattle obtained from the Danish Cattle Research Centre, AU Viborg, Aarhus University, Denmark were analysed. All pregnancies were the result of artificial insemination. The data are included as Additional file 1. These data were recorded systematically and independent of the current study as part of routine herd monitoring. The data included 2734 liveborn singletons born from 10 February 2001 to 19 July 2022.

### Sampling of late-term fetuses

Late-term Holstein fetuses were obtained from cows used for teaching caesarean section to veterinary students at the University of Copenhagen, Denmark. Late-term cows were acquired for this purpose from a Holstein cattle herd and transported to the Large Animal Teaching Hospital before gestation day (GD) 251. Pregnancy had been established through artificial insemination done by a certified technician, who recorded the event directly in the Danish Cattle Database (SEGES Innovation P/S, Aarhus, Denmark). Pregnancy was confirmed by transrectal palpation and recorded in the database.

A caesarean section was performed on sedated cows through a laparotomy on the left side under local analgesia at different time points before the estimated calving date. The calf was euthanised by an intravenous overdose of barbiturate in a jugular vein immediately after extraction from the uterus, while the cow was euthanised after the end of surgery, according to Danish legislation. The procedure was approved by the Danish Animal Experimental Inspectorate (Approval no. 2020-15-0201-00660) and by the local ethics committee.

The extracted fetuses were examined within 2–3 h after euthanasia and before *rigor mortis* had developed. Only singletons were included (n = 54). Firstly, the sex was determined and a brief general assessment of maturity was performed, mainly focusing on development of the hair coat. The general assessment was done by one observer (JSA), who was blinded to the gestation age of the fetus. Each fetus was then weighed and eruption through the gingiva of the three incisors (I1–3) and the canine teeth (CT) was determined. This was done by visual inspection of the vestibular and occlusal surfaces. If eruption was not clearly evident via visual inspection, an acoustic evaluation was carried out by knocking the occlusal surface with the blade of a knife. If the occlusal surface was covered by a mucosa, a dull sound was produced, while a sharp sound was produced if the enamel was not covered by mucosa. In some cases, where the gingiva was pale and difficult to visually differentiate from the enamel, the incisor part of the mandible was removed and placed in an aqueous solution of D&C Red 33 (5-amino-4-hydroxy-3-(phenylazo)-2,7-naphthalenedisulfonic acid; GUM^®^ RED-COTE® Plaque Disclosing Tablets, 1 tablet/100 mL tap water; Sunstar Scandinavia, Askim, Sweden) overnight at 5 °C. The specimen was gently rinsed under tap water the next day, and the gingival borders were inspected. As fetuses were sampled over a seven-year period (2016–2022), not all data were recorded for all fetuses (see Additional file 2 for details). Data on tooth eruption were analysed as eruption present or absent for each tooth, i.e., if any part of the occlusal surface was uncovered by gingival mucosa, the tooth was considered to have erupted.

### Assessment of incisor and canine tooth eruption in neonatal calves

Tooth eruption through the gingival mucosa was assessed in Holstein calves from a production herd within 12 h of birth over a three-month period by manually opening the calf’s mouth and inspecting I1–3 and CT bilaterally. In case of doubt regarding eruption, the occlusal surface was tapped with a steel object and the sound was noted (dull *versus* sharp sound). Tooth eruption was recorded as either present or absent for each tooth as explained above. All assessments were carried out by one observer (MD). In total, 54 calves were examined.

### Statistical analyses

Summary statistics were made to analyse the variation in GL and BW of neonatal calves born at the Danish Cattle Research Centre, as well as the correlation between GL and BW. The statistics for GL included minimum, 0.5th,1st 5th, 25th, 50th, 75th, 95th, 99th and 99.5th percentiles to enable a detailed examination of the tail of the GL distribution. The summary statistics were stratified by sex of the calf (male or female) and parity of the dam (1st, 2nd, 3rd and ≥ 4th), as well as given overall. We arbitrarily used the 0.5th and 99.5th percentiles to exclude potential recording errors and pathological gestations (preterm delivery and prolonged gestation).

Thresholds for exceeding G90 were based on the summary statistics. The thresholds were calculated as the statistic in question (e.g., the median) × 0.9. For example, a GL of 278 days would result in a G90 threshold of 278 × 0.9 = 250 days. These thresholds were used for further analyses.

The correlation between GL and body weight was estimated using linear regression, adjusted for parity group of the dam (1, 2, 3, or ≥ 4) and sex of the neonatal calves (male or female).

The probability of exceeding the G90 threshold given a specific BW or eruption of specific teeth (none, I1, I2, I3 and canine teeth) was estimated using logistic regression with the following formula:

P(GL > G90_*i*_ | predictor) = intercept + predictor,where GL was the observed GL, G90_*i*_ was the threshold based on the *i*th summary statistic, where *i* included the median, the 3rd quartile, the 95th percentile, the 99.5th percentile and the maximum GL in the data from the research herd, and predictor could be either BW or number of erupted teeth (I1–3, CT). The logistic regression was done using the glm-function in R [7].

## Results

### Gestation length of Holstein cattle

Analyses of the overall data based on 2734 Holsteins giving birth to a single liveborn fetus revealed a median GL of 278 days with a minimum of 239 days, a maximum of 317 days and an interquartile range of 275–281 days. The analysis showed that 99% of the dams gave birth between 261 and 290 days of gestation. The GL was affected by parity and fetal sex (Table 1).

**Table 1:** Summary statistics of calculated gestation length for 2,734 Holsteins giving birth to a liveborn singleton

Sex of offspring	Parity	n	Gestation length (days)										
			Min	P0.5	P1	P25	P50	P75	P95	P97.5	P99	P99.5	Max
Overall		2,734	239	259	261	275	278	281	286	288	290	292	317
Female	1	540	247	259	264	273	277	279	284	285	287	287	317
Female	2	412	239	252	256	274	277	280	285	287	289	294	296
Female	3	250	240	262	267	276	278	282	287	288	289	290	292
Female	≥ 4	150	256	263	266	275	278	282	286	292	292	293	296
Male	1	542	251	260	266	275	278	281	285	286	288	289	291
Male	2	414	253	260	261	276	280	283	288	289	291	294	306
Male	3	256	259	260	262	277	280	282	288	289	291	292	294
Male	≥ 4	170	261	269	272	277	280	283	288	289	291	292	292

Data showing the association between calculated gestation length and the parity of the dam and sex of the offspring are included. P = percentile

### Body weight of neonatal Holstein calves

**Fig. 1:**
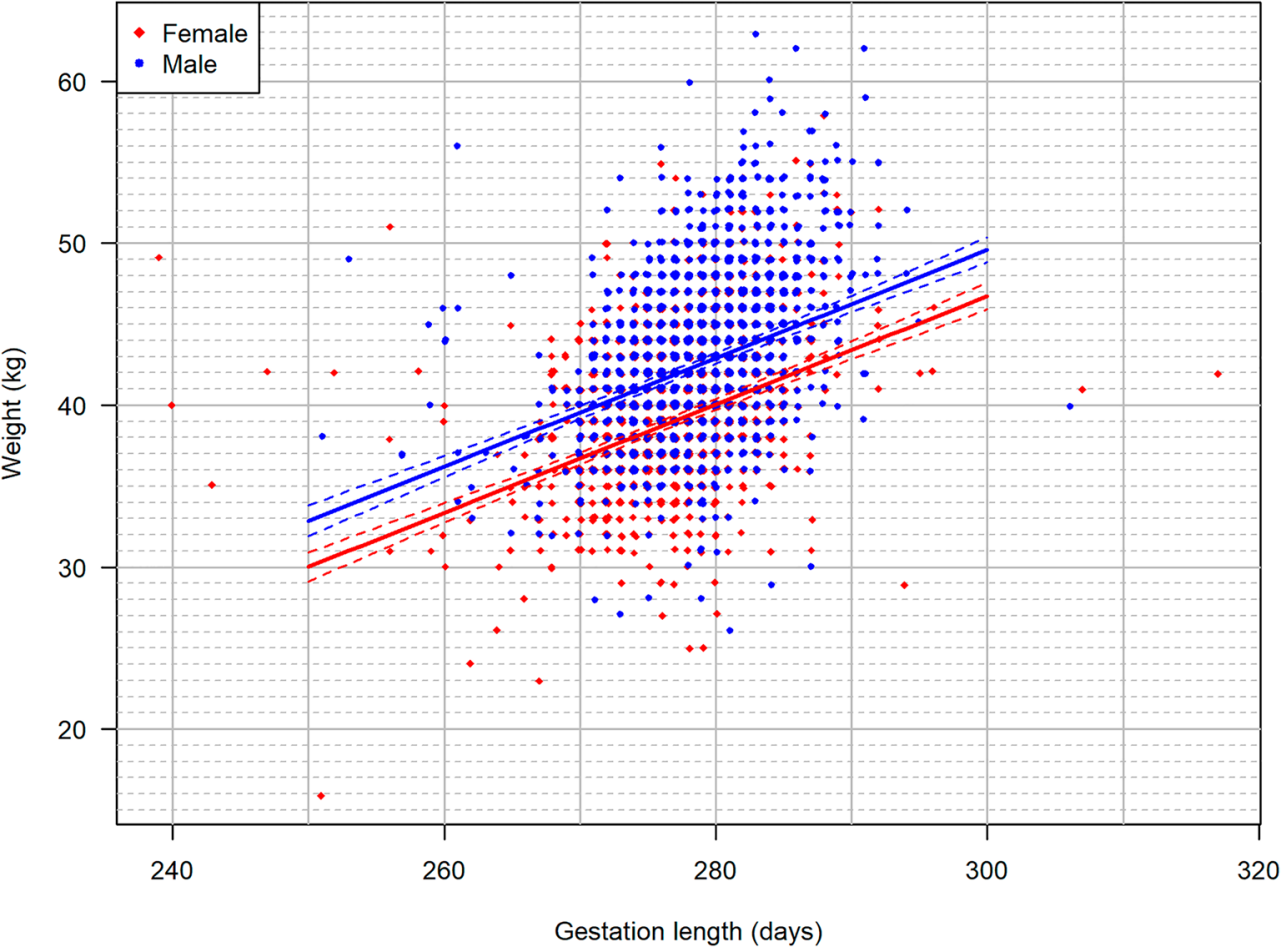
Scatterplot of body weight as a function of gestation length (GL). Data based on 2734 liveborn singleton Holstein calves born at the Danish Cattle Research Centre in the period 2001–2022. The size of the dots increases with increasing number of calves. The two lines show the linear relationship between GL and body weight, while the dashed lines mark the 95% confidence limits of the means for a given GL for males and females, respectively

Analysis of the overall BW data from the 2734 liveborn singletons gave a median BW of 42 kg with a minimum of 16 kg and a maximum of 63 kg. The BW depended on parity and fetal sex, i.e., the median BW ranged from 39 to 41 kg for females and from 42 to 45 kg for males (Table 2). Results of the linear regression analysis suggested an R^2^ = 0.27 (95% confidence interval (CI) 0.25–0.30) (Fig. 1, Additional file 3), with male calves almost 3 kg heavier than female calves, and first-parity calves almost 2 kg lighter than calves born to higher parity cows.

**Table 2:** Summary statistics of the body weight of 2734 liveborn Holstein singletons

Sex of offspring	Parity	n	Body weight (kg)										
			Min	P0.5	P1	P25	P50	P75	P95	P97.5	P99	P99.5	Max
Overall		2,734	16	29	30	38	42	45	51	53	55	56	63
Female	1	540	16	29	29	36	39	42	46	48	50	52	54
Female	2	412	24	27	29	38	41	44	49	50	52	54	55
Female	3	250	23	29	30	38	41	45	52	52	54	55	58
Female	≥ 4	150	25	29	31	38	41	44	48	50	51	52	54
Male	1	542	26	28	30	39	42	45	51	52	55	56	63
Male	2	414	28	32	33	41	44	48	54	55	56	58	60
Male	3	256	30	31	33	42	45	48	53	55	58	61	62
Male	≥ 4	170	33	35	35	41	45	48	52	54	54	56	59

Data showing the association between body weight and the parity of the dam and sex of the offspring are included. P = percentile

### General appearance and body weight of preterm Holstein fetuses

**Fig. 2:**
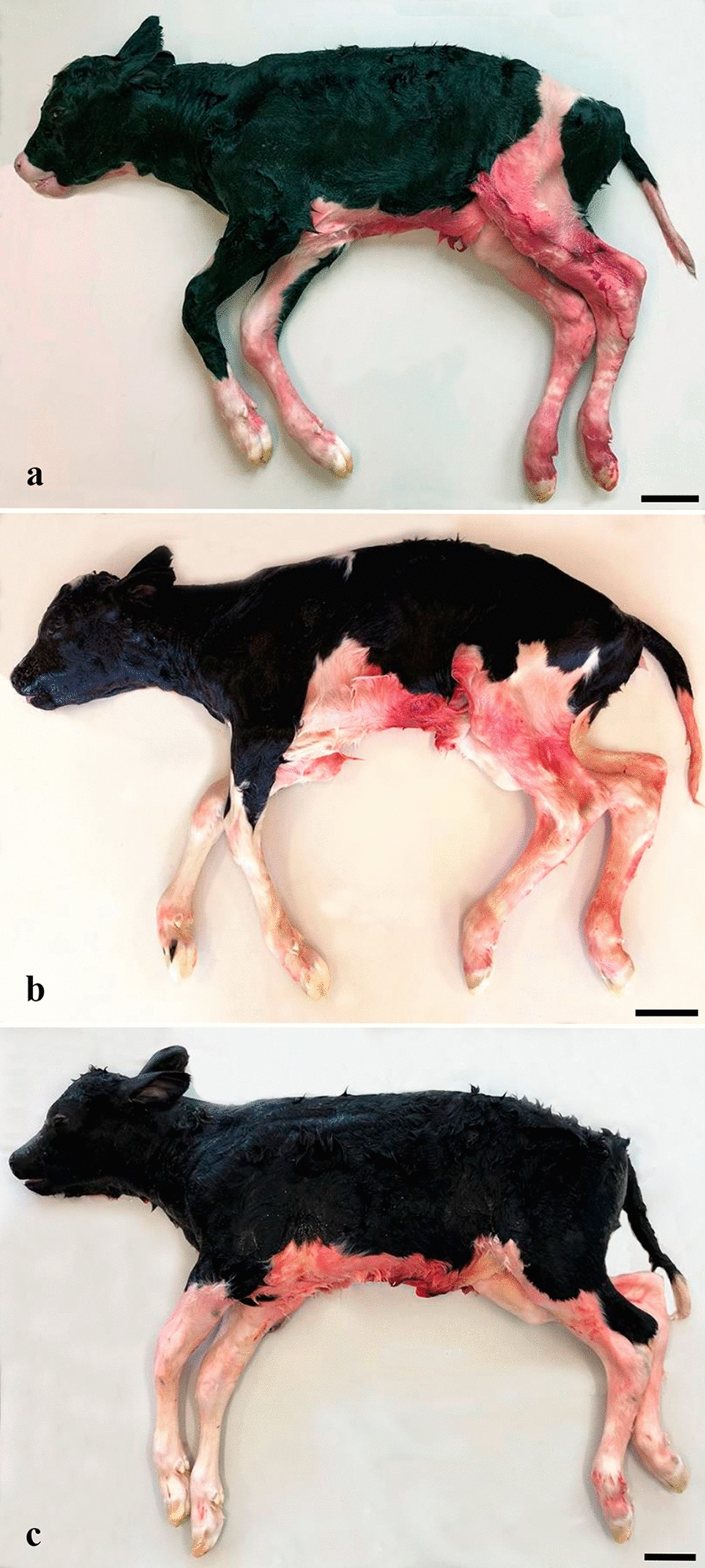
Gross appearance of Holstein fetuses during late gestation. **a**–**c** Holstein fetuses delivered by caesarean section on gestation day 253 (**a**), 260 (**b**) and 273 (**c**). The fetuses differed in body weight (23.0, 33.7 and 52.5 kg for fetus **a**, **b** and **c**, respectively) and crown rump length (96, 95 and 104 cm for fetus **a**, **b** and **c**, respectively). When directly compared to each other, fetus **a** and **b** appear more immature than fetus **c**; an observation that may be biased by the smaller size of these two fetuses, as it is difficult to define what the “premature appearance” represents as the fetuses are otherwise fully developed

**Fig. 3:**
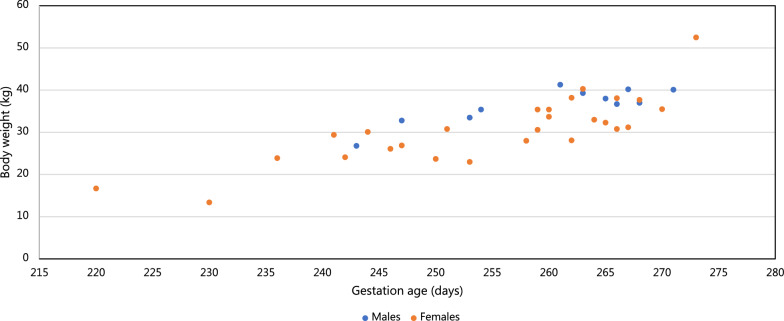
Diagram showing the correlation between age and body weight of late-term Holstein fetuses (11 males and 27 females). The body weight increases during late gestation with a tendency of males being heavier than females. However, as can been interpreted by the weight distributions, body weight varies considerably as for neonatal calves (see Fig. 1)

Examination of the fetuses from the caesarean sections (n = 54) showed that all cases in this sample of late-term fetuses had the overall appearance of a mature fetus (Fig. 2). It was not possible for the observer (JSA) to determine whether the fetuses were above or below the G90 threshold based on a visual assessment of their general appearance. The overall fetus size, as reflected in the BW, increased with increasing GL. However, when assessing a specific BW, this corresponded to a wide GL interval, e.g., a BW of 30 kg covered a GL interval from 244 to 266 days (Fig. 3).

### Eruption of the incisors

**Fig. 4:**
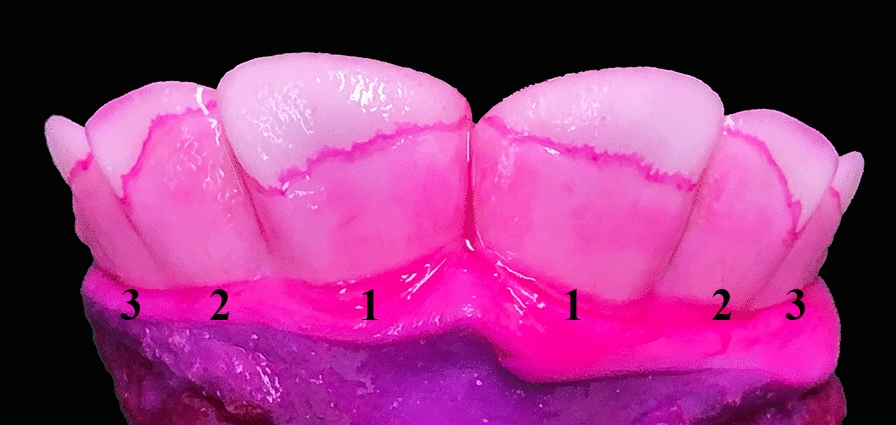
Complete eruption of incisor teeth 1–3 through the gingival surface in a 264-day-old Holstein fetus. In most cases, eruption of the incisor teeth is symmetrical in terms of sequence, eruption site and degree of eruption. Penetration of the gingiva starts at the lateral angle of the occlusal surface for incisors 1 and 2, and is more advanced laterally. Eruption of incisor 3 starts at the highest point of the occlusal surface, which is located at the centre (see Fig. 5b). The gingival mucosa still covers part of the enamel, as clearly identified by intense pink staining of the gingival border. Anterior part of the hemimandibles stained with D&C Red 33

**Fig. 5:**
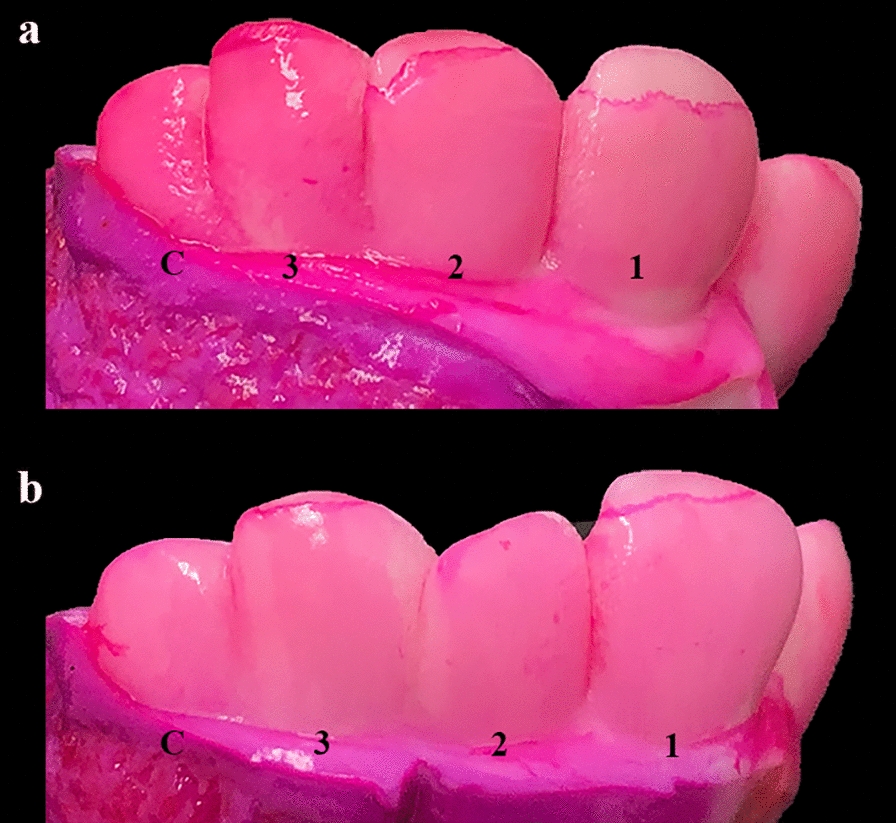
Sequence of incisor tooth eruption. Part **a** shows eruption of incisors 1 and 2. The most medial incisor tooth (incisor 1) is the first incisor to erupt through the gingival surface. Penetration of the gingiva starts at the lateral angle of the occlusal surface. In most cases, this is followed by eruption of incisor 2, also penetrating at the lateral angle. Part **b** shows eruption of incisor 1, but in this case, incisor 3 has erupted before incisor 2, which is still covered by the gingival mucosa. The figure also shows that the sequence of incisor tooth eruption varies between cases as both specimens originate from a 253-day-old Holstein fetus. Anterior part of the right hemimandible stained with D&C Red 33

Eruption of I1 and I2 started at the lateral angle of the occlusal surface and then progressed medially and ventrally, while the eruption of I3 started centrally in the occlusal surface and then expanded laterally, medially and ventrally. The enamel of the anterior surface was exposed in a highly symmetrical and consistent way when the eruption was advanced (Fig. 4), while this varied for less advanced eruption (Fig. 5). Incisor 1 always erupted before the other two incisors, while the eruption time and extent of enamel exposure varied for I2 and I3, although the general pattern was that I2 erupted before I3 and the eruption was symmetrical in terms of both eruption time and degree of enamel exposure. However, there were cases where I3 erupted before I2, where the left or the right incisor had erupted before the corresponding contralateral incisor or where the degree of enamel exposure was asymmetrical.

**Fig. 6:**
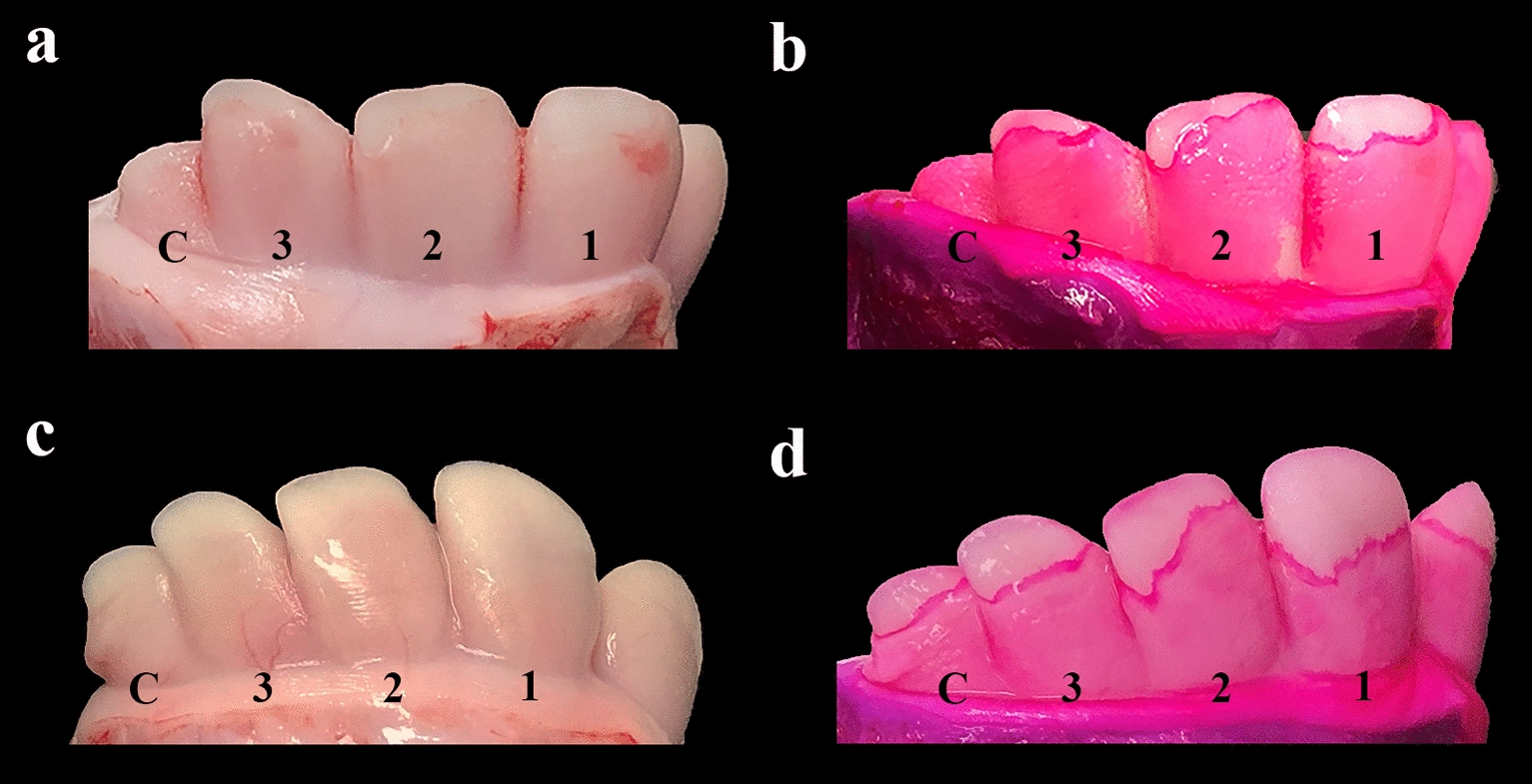
Unstained hemimandibular specimens compared to specimens stained with D&C Red 33. Anterior part of the right hemimandible of bovine fetuses. Tooth eruption through the gingival mucosa is difficult to assess in some fetuses, especially in cases with a pale gingiva. Staining with D&C Red 33 overnight enables accurate identification of the gingival margin, which is stained an intense shade of pink (**b**, **d**). Specimens **a**–**b** originate from a 270-day-old Holstein fetus, while c–d originate from a 264-day-old fetus. Notice that the 264-day-old fetus has complete eruption of all incisor teeth and canine teeth, while this has not yet occurred in the other fetus despite it being 6 days older (**a**–**b**). The findings from the fetus shown in c–d emphasise that complete eruption of all incisor teeth and canine teeth may occur close to the 251-day gestational age limit after which transport of the dam is prohibited. Note that the eruption is advanced in this case

Visual assessment of tooth eruption was challenging in cases where the gingiva was pale and with poor contrast to the enamel, and in cases where eruption was only in the upper margin of the occlusal surface. Tapping the occlusal surface with a metal object, e.g., the blade of a knife, was a simple and useful method to identify eruption. For documentation (e.g., in forensic veterinary medicine), staining of the specimen with D&C Red 33 proved to be highly useful as the gingival mucosal border was stained an intense shade of pink, thus facilitating visual inspection (Fig. 6).

The first incisor had erupted in all fetuses older than 236 days (n = 49, GL range [GLR] 236–284 days), while it had not erupted in younger fetuses (n = 3; GLR 220–235 days). The second incisor had bilaterally erupted in all fetuses older than 241 days (n = 39; GLR 241–284 days). One 241-day-old fetus had unilateral eruption, while eruption of I2 had not occurred in 12 fetuses (GLR 220–259 days). For I3, eruption was observed in fetuses aged 236–281 days (n = 43), while eruption had not occurred in 11 fetuses ranging in age from 220 to 272 days.

When evaluating tooth eruption after grouping fetuses into age intervals of 10 days (Table 3), 44% of fetuses in the age interval 241–250 days had eruption of at least one of each incisor 1–3. For older fetuses, 97% had eruption of at least one of each incisor 1–3. Assessment of incisor tooth eruption in the 54 Holstein calves born in the herd study and examined within 12 h after delivery revealed eruption of all incisors.

**Table 3:** Summary data on observed eruption of incisor and canine teeth in Holstein fetuses (n = 52) and neonatal calves (n = 54)

Fetal age (days)	No. of fetuses	Eruption^§^ of incisor no				Eruption of all incisors
		1	2	3	C	
**≤ 230**	2	0	0	0	0	0/2
**231–240**	2	1	0	1	0	0/2
**241–250**	9	9	4	4	0	4/9
251–260	12	12	12	12	1	12/12
261–270	20	20	20	20	4	20/20
271–280	6	6	6	5	1	5/6
≥ 281	1	1	1	1	0	1/1
Neonatal	54	54	54	54	22	54/54

Bold indicates bovine fetuses from dams pregnant up to gestation day 250 (thus permitted to be transported according to the current European legislation based on an expected GL of 278 days)

I1: Incisor 1, I2: Incisor 2; I3: Incisor 3; C: Canine tooth; ^§^Tooth recorded as “erupted” if at least one of the two corresponding teeth had erupted, i.e., either the left or the right I1 or both

### Eruption of the canine teeth

Eruption of the CT was not observed before gestation day 259 for fetuses delivered by caesarean section. In general, the CT had erupted in only a small number of fetuses, while 46% of the neonatal calves had an erupted CT (Table 3).

### Statistical analysis of the association among the G90 threshold, body weight and tooth eruption

**Fig. 7:**
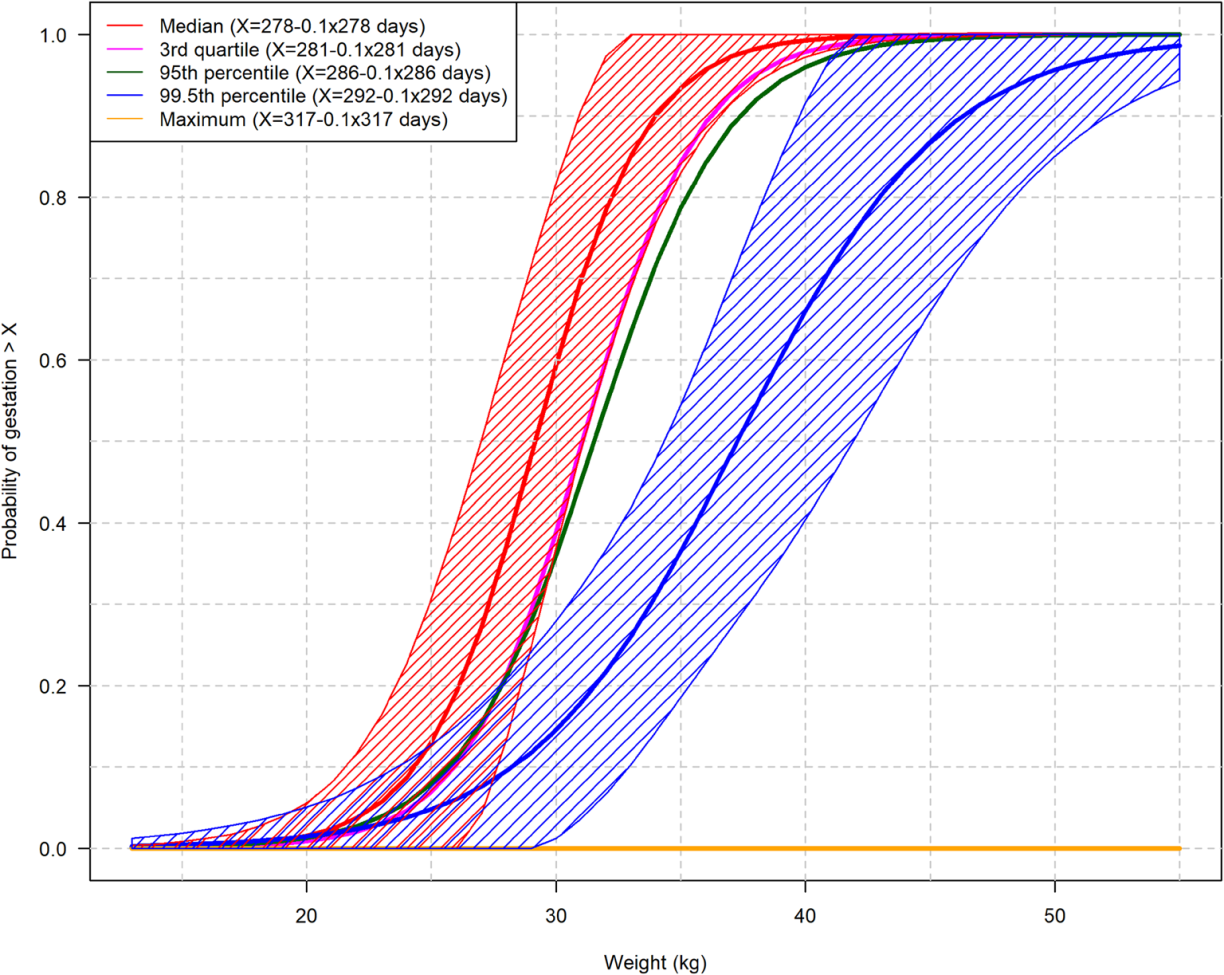
Correlation between body weight and probability of fetal age exceeding the gestation length (G90) threshold of 250 days, i.e., 278 days—10%, where 278 is the overall median gestation length for Holstein cattle. The figure shows the median, 3rd quartile, 95th percentile, 99.5th percentile and maximum based on the data presented in Table 1. The 95% confidence interval (CI) (shaded) is shown only for the median (red) and 99.5th percentile (blue). The figure can be used to predict the probability of a Holstein fetus with a given body weight exceeding the G90 threshold based on a median GL of 278 days. For example, if a fetus has a body weight of 35 kg, the probability of the fetus exceeding G90 is 0.93 (95% CI 0.83–1.00) based on the median, and 0.37 (95% CI 0.19–0.54) based on the 99.5th percentile

Using the calculated GL median of 278 days (Table 1), we determined the G90 threshold to be day 250. The outcome of the logistic regression analysis of the correlation between the G90 threshold and BW is shown in Fig. 7 and includes the median, 3rd quartile, 95th percentile, 99.5th percentile and maximum, as well as the 95% confidence interval for the median and 99.5th percentile.

**Fig. 8:**
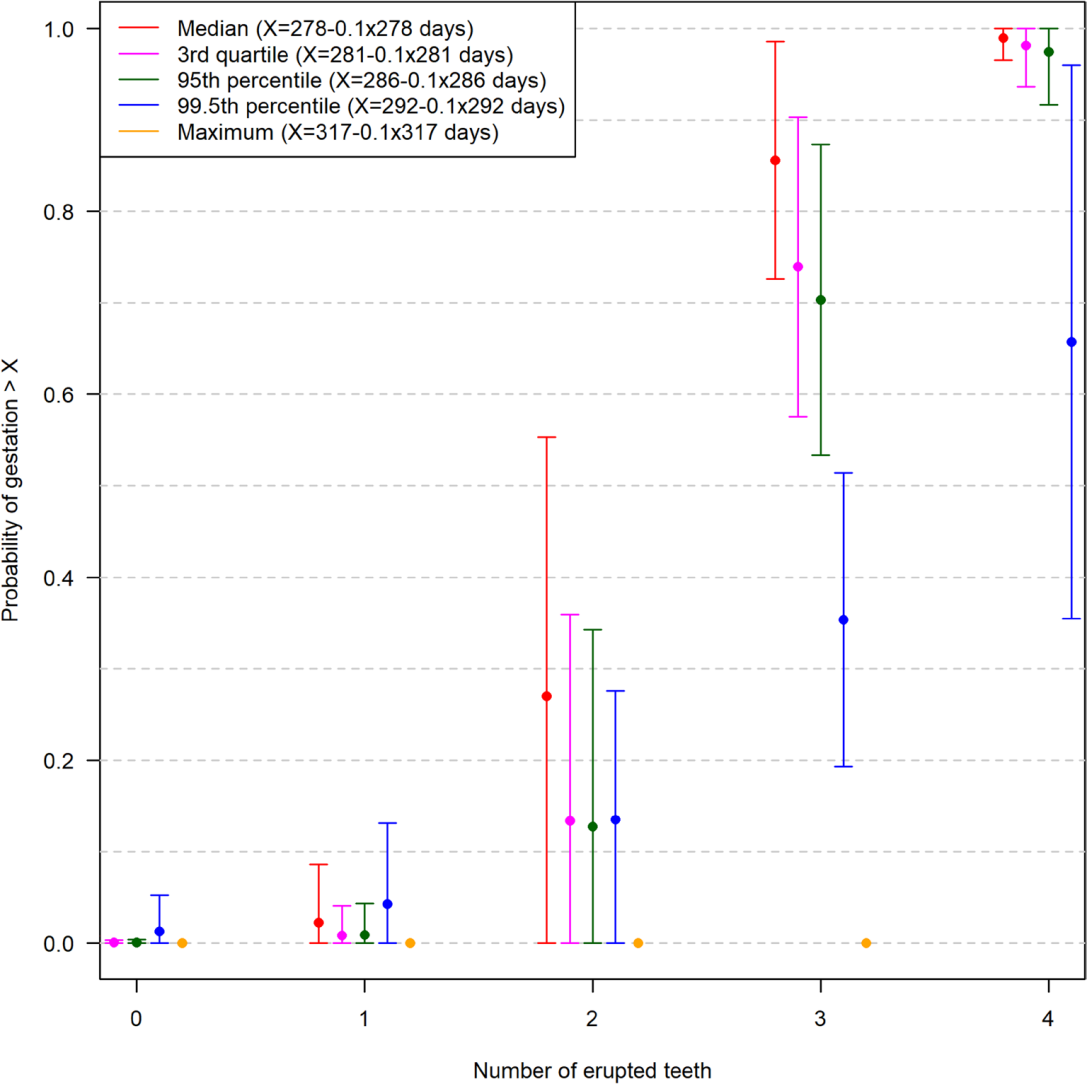
Association between pairwise eruption of incisor teeth (I) 1–3, canine teeth (CT) and probability of fetal age exceeding the gestation length (G90) threshold of 250 days, i.e., 278 days—10%, where 278 is the overall median gestation length for Holstein cattle. Gestation length data are based on the data presented in Table 1. A jitter has been used to separate the graphs for ease of readability. The error bars illustrate the 95% confidence interval. It would often be the case that 1 would correspond to I1, 2 to I2, 3 to I3 and 4 to CT, as this is the most common sequence of eruption (see text), but when using the figure to determine fetal age, it is important to assess the number of pairwise tooth eruptions without regard to their anatomical order. The figure can be used to predict the probability that a Holstein fetus with a given pairwise sequence of eruption of the I1–3 and CT will exceed the G90 threshold based on a median GL of 278 days. For example, if a fetus has an eruption of I1–3, the probability of the fetus exceeding G90 is around 0.86 (95% CI 0.72–0.99) based on the median, and 0.71 (95% CI 0.53–0.88) based on the 99.5^th^ percentile

The association between the G90 threshold and eruption of I1–3 and CT was calculated in a similar way. The outcome of the logistic regression analyses including CIs are shown in Fig. 8. The first teeth to erupt in a pair (I1, I2, I3 and CT) in individual fetuses are presented. The probability that a fetus with a given number of erupted teeth had exceeded the G90 threshold (day 250) was as follows: one erupted tooth (typically I1): 0.02 (95% CI 0–0.09), two erupted teeth: 0.27 (95% CI 0–0.55), three erupted teeth: 0.86 (95% CI 0.73–0.99) and four erupted teeth: 0.99 (95% CI 0.97–1.00). If the 95th percentile was used instead, these probabilities were a few percentage points lower, while they were much lower if the 99.5th percentile was used, e.g., the probability of three erupted teeth was 0.35 (95% CI 0.19–0.51) (Fig. 8).

## Discussion

Analyses of the GL data showed a wide variation in GL, as also reported by others [2–5]. Using the P0.5 and P99.5 values (i.e., GL of 261 days and 290 days), the dams passed the G90 threshold at gestation day 235 and day 261, respectively, i.e., within an interval of 26 days. The wide variation in GL therefore had a major impact on when the dams entered the last 10% of gestation. Given that the influence of fetal sex and the dam’s parity amounted to ± 2 days (Table 1), their impact when calculating the GL for compliant transport of pregnant females was not significant, as these only added around ± 0.2 days to the GL. The impact of environmental factors, sire, etc. on GL has been reported to be 1–2 days [2] and will therefore cumulatively impact the G90 threshold by less than a day. The wide interval during which Holsteins enter the last 10% of gestation makes it challenging to provide forensic evidence that a particular cow was transported after passing the G90 threshold based on general population-based breeding data, unless the animal expresses signs of imminent calving such as relaxation of the pelvic ligaments [8].

The overall BW of neonatal Holstein calves varied from 28 to 56 kg with a mean of 42 kg. The BW was affected by the sex of the calf, with male calves generally being heavier than female calves, and by the dam’s parity, with fetal BW increasing with increasing parity, especially for male calves (Table 2). These findings correspond to earlier reports [2–5]. Analysing the association between the GL of the dam and the BW of the offspring revealed an R^2^ of only 0.27. The BW of the late-term fetuses delivered by caesarean section also varied considerably (Fig. 3). The wide variation in BW of late-term fetuses as well as neonatal calves makes it challenging to use a specific BW as forensic evidence for a fetus having exceeded the G90 threshold. As an example, the BW of neonatal female calves delivered by Holstein heifers varied from 28 to 56 kg, i.e., by 100% (Table 2), and similarly wide BW intervals were found for other parities. Another example shows that the BW of male calves born after a GL of 270 days varied from 32 to 43 kg. The wide range in BW was also reflected in the BW of preterm fetuses, but reliable statistical data could not be provided for these due to their low numbers (n = 54) and variation in GL. However, it is likely that the wide variation in BW found in the neonatal calves would also have been observed in late-term fetuses if a large enough sample had been available.

The Danish regulations for defining the G90 threshold are based on the average gestation period for the relevant animal species. The average GL for cattle is set to 282 days [9], which is higher than found in this study based on calving data from 2734 Holsteins at the Danish Cattle Research Centre. This suggests that using an average GL across all cattle breeds and without taking into account the extensive natural variation in GL is not applicable to forensic veterinary medicine.

In suspected cases of violation of the transport ban for late-term pregnant cattle, the breeding date may not be available to the veterinary authorities, e.g., due to legal protection of herd data or a lack of detailed breeding records. Under such circumstances, it would be useful to provide probability data for a specific animal having passed the G90 threshold based on e.g., fetal BW. In our study, we used the calculated median GL of 278 days for Holsteins to illustrate the probability of the dam exceeding the G90 threshold at a given BW. We also provided illustrations for the 3rd quartile, 95th percentile, 99.5th percentile and maximum GL, including 95% CIs for the median and 99.5^th^ percentile GL to illustrate the uncertainties in the available fetal age assessment. These data can be used to assess the probability of the dam having passed the G90 threshold at a given fetal BW.

We have previously demonstrated considerable variation in the time that morphological characteristics appear in the developing bovine fetus [6], but the data in that study almost entirely originated from Holstein fetuses before the G90 threshold. In the present study, we assessed the overall appearance of the fetus and eruption of incisor and canine teeth in premature calves delivered by caesarean section. This approach was chosen as these parameters have formed the basis for prosecuting owners of pregnant cows or heifers in Denmark when a late-term bovine pregnancy has been detected at post mortem inspection at an abattoir (JSA, personal observation). Our data show that the incisor teeth erupt through the gingival mucosa in an almost consistent pattern, starting with I1 followed by I2, I3 and CT. Eruption was usually bilaterally symmetrical, but a variation in sequence and symmetry was found in some cases. The prevalence of eruption of all incisors increased with increasing fetal age, however, all incisors had erupted in around 50% of fetuses aged 241–250 days (Table 3), i.e., below the G90 threshold if based on a median GL of 278 days. The three pairs of incisors had erupted in all neonatal Holstein calves (n = 54). It should however be noted that the data were based on only 54 late-term fetuses. Analysis of a large number of fetuses would have provided a more accurate estimate.

Eruption of the CT was not observed before gestation day 259 and occurred late in gestation (Table 3) or during the postnatal period in many calves, as only 46% of the examined neonatal calves in the herd data set had erupted CT. When analysing tooth eruption in relation to GL in a similar way to BW, the results of the logistic regression showed that the 95% CI was very wide when only I1–2 or I1–3 had erupted through the gingival mucosa, thus confirming the uncertainty associated with using eruption of these teeth as a definitive criterion for exceeding the G90 threshold. When considering the eruption of all teeth, the 95% CI was narrow with the exception of the 99.5th percentile. These findings indicate that eruption of the I1–3 and CT can be used as a forensic hallmark to identify fetuses that have passed the G90 threshold. However, the CT will not have erupted for many fetuses exceeding this threshold. Basing prosecution on the eruption of CT may therefore raise legal concerns.

## Conclusion

Forensic age assessment of late-term bovine fetuses is challenging. Analysis of breeding data from 2437 Holsteins each giving birth to a single liveborn calf revealed a wide variation in GL, thereby illustrating the difficulties faced when defining the G90 threshold for pregnant cows or heifers that do not reach parturition, e.g., due to slaughter. The BW of late-term fetuses as well as neonatal calves varied significantly. As a result, BW is not a reliable parameter for identifying fetuses that have exceeded the G90 threshold, nor is the overall appearance of the fetus. Statistical analysis of the association between fetal age and eruption through the gingival mucosa of I1–3 and CT revealed significant variation, making tooth eruption a challenging parameter to use in forensic cases. Assessment of the evaluated parameters, therefore, cannot be considered a scientifically validated method to conclude definitively and beyond reasonable doubt whether or not a given fetus has passed the G90 threshold.

## Supplementary Information

**Additional file 1: **Data on gestation length (GL), parity, sex and fetal body weight (BW) at delivery from 2,734 Holstein singletons born at the Danish Cattle Research Centre, Aarhus University, Denmark from 10 February 2001 to 19 July 2022. Excel file.

**Additional file 2: **Number of fetuses from caesarean sections with recorded data.

**Additional file 3: **Regression of body weight at birth on gestation length (GL), parity and sex of 2,737 neonatal liveborn Holstein singletons. Model fit: R^2^=0.27 [95% confidence interval: 0.25–0.30].

## Data Availability

Most data are included as supplementary materials. Other datasets used and/or analysed during the current study are available from the corresponding author on reasonable request.

## Abbreviations

AR: Age range
CI: Confidence interval
CT: Canine tooth/teeth
G90: The last 10% of the gestation period
GL: Gestation length
GLR: Gestation length range
I1: Incisor tooth/teeth 1
I2: Incisor tooth/teeth 2
I3: Incisor tooth/teeth 3
