# Supplementary material for: Forensic age assessment of late-term bovine fetuses
Source: Acta Vet Scand. 2023 Jun 24;65:27. doi: 10.1186/s13028-023-00691-0 (PMC10290400; doi:10.1186/s13028-023-00691-0)
Supplement: Supplementary file 2 — Additional file 2: Number of fetuses from caesarean sections with recorded data. [file 13028_2023_691_MOESM2_ESM.docx]

**Additional file 2.** Number of fetuses from caesarean sections with recorded data

| **Data recorded** | **Singletons** | **Twins**^#^ |
| --- | --- | --- |
| Total number of fetuses | 54 | 4 |
| Number of fetuses with recorded tooth eruption | 52 | 4 |
| Number of fetuses with recorded body weight | 40* | 4 |
| Number of fetuses with recorded sex | 38* | 4 |

* These groups consist of the same fetuses; ^#^ The figure represents the number of fetuses.
