# Supplementary material for: Forensic age assessment of late-term bovine fetuses
Source: Acta Vet Scand. 2023 Jun 24;65:27. doi: 10.1186/s13028-023-00691-0 (PMC10290400; doi:10.1186/s13028-023-00691-0)
Supplement: Supplementary file 3 — Additional file 3: Regression of body weight at birth on gestation length (GL), parity and sex of 2,737 neonatal liveborn Holstein singletons. Model fit: R2=0.27 [95% confidence interval: 0.25–0.30]. [file 13028_2023_691_MOESM3_ESM.docx]

**Additional file 3.** Regression of body weight at birth on gestation length (GL), parity and sex of 2,737 neonatal liveborn Holstein singletons. Model fit: R^2^=0.27 [95% confidence interval: 0.25–0.30].

| **Variable** | **Estimate** | **Std. error** | **P-value** |
| --- | --- | --- | --- |
| (Intercept) | -54.76 | 4.49 | <0.0001 |
| GL | 0.34 | 0.02 | <0.0001 |
| Parity = 2 | 1.86 | 0.21 | <0.0001 |
| Parity = 3 | 2.09 | 0.24 |  |
| Parity = 4 | 1.60 | 0.29 |  |
| Sex = male | 2.85 | 0.17 | <0.0001 |
